# Genome Sequencing, Assembly, and Characterization of *Cyberlindnera rhodanensis J52* as a Non-*Saccharomyces* Yeast with Ester-Enhancing Potential

**DOI:** 10.3390/jof11020135

**Published:** 2025-02-11

**Authors:** Yong Shen, Zongcai Tu, Lizhou Tang, Yiyong Luo

**Affiliations:** 1 National R&D Center for Freshwater Fish Processing, College of Life Sciences (Health), Jiangxi Normal University, Nanchang 330022, China; shenyong@jxnu.edu.cn (Y.S.); 004756@jxnu.edu.cn (Z.T.); tanglizhou@163.com (L.T.); 2 State Key Laboratory of Food Science and Resources, Nanchang University, Nanchang 330047, China

**Keywords:** *Cyberlindnera rhodanensis*, non-*Saccharomyces*, isoamyl acetate, ester, selenoprotein

## Abstract

*Cyberlindnera rhodanensis* J52, a non-*Saccharomyces* yeast isolated from edible roses, markedly improves the organoleptic qualities of fermented foods. To facilitate the development and application of this strain, this study sequenced and assembled the genome of *C. rhodanensis* J52, subsequently conducting functional annotation of its genes utilizing the NR, Swiss-Prot, COG, GO, KEGG and CAZy databases. The findings revealed that this yeast harbors genes involved in the biosynthesis of flavor compounds, including higher alcohols, acetate esters, ethyl esters, volatile organic acids, aromatic amino acids and benzyl alcohol or benzaldehyde. Furthermore, it possesses β-glucosidase, an extracellular enzyme which enhances the flavor profile of fermented products. Further analysis revealed that the yeast features biosynthetic pathways for the production of isoamyl acetate, isoamyl 2-methylbutyrate, benzyl acetate, phenethyl acetate, ethyl butanoate and ethyl decanoate, which verifies its ability to produce esters at the genetic level. Additionally, the yeast was found to have the capacity to biosynthesize selenoproteins, suggesting that it not only enhances flavor but also imparts functional benefits. These findings provide a theoretical foundation for the further exploration and application of *C. rhodanensis* J52.

## 1. Introduction

*Cyberlindnera rhodanensis*, a type of non-*Saccharomyces* yeast, has garnered increasing interest within the fermentation industry due to its capacity to secrete distinctive enzymes, produce desirable secondary metabolites and generate unique aroma compounds. For instance, *C. rhodanensis* shows potential for industrial processes aimed at converting lignocellulose into xylitol [1]. The diverse array of enzymes secreted by *C. rhodanensis* further underscores its significant applicability within the fermentation industry. *C. rhodanensis* strains A22.3 and A45.3, isolated from miang, have exhibited the capacity to produce tannase, an enzyme beneficial for the extraction and bioconversion of tea catechins [2,3]. Additionally, *C. rhodanensis* demonstrated the ability to degrade phytic acid, an anti-nutritional factor [4]. *C. rhodanensis* DK, isolated from Laphet-so, produces an extracellular thermostable β-glucosidase, which is crucial not only for cellulose hydrolysis but also for the release of aroma compounds in fruits and fermented products [5,6]. Furthermore, the co-fermentation of *C. rhodanensis* with other strains to generate consumer-preferred fermented products is an emerging area of research. For example, co-fermentation with *Lactobacillus pentosus* facilitates the conversion of cashew apple waste into a low-alcohol healthy beverage [7]. Our previous study also showed that co-fermentation with *Pediococcus pentosaceus* significantly influences the microbial community and volatile compounds in fermented peppers, thereby enhancing their sensory attributes [8]. However, despite its considerable potential, the molecular underpinnings of these properties in *C. rhodanensis* remain inadequately explored.

Currently, a diverse array of omics methodologies exists to elucidate the roles and mechanisms of strains in the fermentation process, yet genomics remains the foundational technology upon which other omics are built. However, genomic data about non-*Saccharomyces* yeasts remains scant in comparison with those of *Saccharomyces cerevisiae*, and the genomic insights for *C. rhodanensis* are particularly limited. To fully comprehend the application potential of this strain, it is imperative to sequence and conduct a thorough analysis of its entire genome.

*C. rhodanensis* J52, previously isolated from edible roses in our laboratory, has been identified for its potential to enhance the flavor profile of fermented products [8]. However, the lack of genomic information for this strain hinders a deeper understanding and its application in industrial contexts. Therefore, this study aimed to sequence the genome of the strain and annotate its composition and function using various databases while also predicting the metabolic pathways responsible for the production of volatile flavor compounds.

## 2. Materials and Methods

### 2.1. Strain and Cultural Condition

*C. rhodanensis* J52 was isolated from edible roses in our previous study [8]. The strain was cultured in YPD medium at 30 °C and 150 rpm for 24 h.

### 2.2. Genome Sequencing, Assembly and Annotation

The genome of *C. rhodanensis* J52 was sequenced using the Illumina and PacBio Sequel platform (Shanghai Majorbio Co., Ltd., Shanghai, China). Then, the genome sequence was assembled de novo from the sequencing data using flye V2.9.2 [9]. After genome assembly, protein-coding genes (CDSs), tRNAs, rRNAs and repeated sequences were predicted using Maker2, tRNAscan-SE, barrnap (https://github.com/tseemann/barrnap/) and RepeatMasker (http://www.repeatmasker.org/), respectively [10,11]. Functional annotation of CDSs was performed by searching against databases, including NR, Swiss-Prot, COG, KEGG and GO. Secondary metabolite biosynthesis gene clusters in the *C. rhodanensis* J52 genome were identified by anti-SMASH 7.0 [12].

### 2.3. Analysis of Carbohydrate-Active Enzymes (CAZymes)

The genes encoding CAZymes were predicted using the CAZy database and the software Diamond version 0.8.35 and hmmscan version 3.1b2, February 2015. These genes were then functionally annotated using the CAZy, NR, Swiss-Prot and SignalP databases.

### 2.4. Analysis of Genes and Pathways Involved in Flavor Production

Referring to the work of Giorello et al. [13], the genes involved in the synthesis of flavor compounds, such as higher alcohols, ethyl esters, acetate esters, volatile organic acids, aromatic amino acids and benzyl alcohol and benzaldehyde in the *C. rhodanensis* J52 genome, were to be identified. Briefly, we downloaded these genes from the *Saccharomyces* Genome Database (SGD; http://www.yeastgenome.org/) and performed BLAST analysis against the *C. rhodanensis* J52 genome using TBtools [14]. The synthesis pathways of esters were predicted based on the BLAST results of related genes and KEGG pathway annotations. The descriptions of these genes are shown in Table S1.

### 2.5. Prediction of Selenoprotein Biosynthetic Pathways

The biosynthesis pathway for selenoprotein in *C. rhodanensis* J52 was predicted based on the functional annotation of the J52 genome and existing references about selenocompound metabolism [15,16,17].

## 3. Results

### 3.1. C. rhodanensis J52 Genome’s General Features

**Figure 1:**
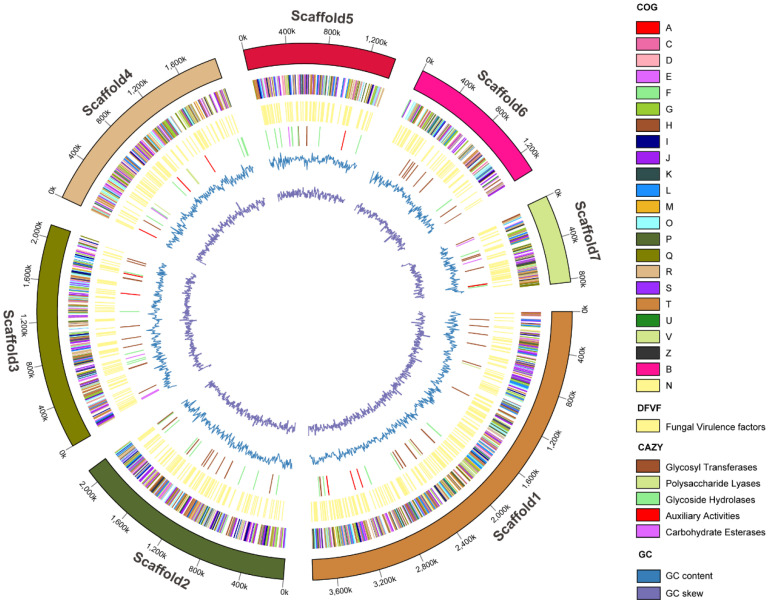
Circular diagram of the *C. rhodanensis* J52 genome.

The genome of *C. rhodanensis* J52 was sequenced and yielded 13,736,238 bp with an average GC content of 51.49%. The genome harbors 4563 CDSs with an average length of 2247.05 bp and 208 RNAs (175 tRNAs and 33 rRNAs) (Table 1). The 175 tRNA encoding sequences corresponded to all 20 standard amino acids as well as the unusual amino acid selenocysteine (Table S2). Additionally, approximately 4.52% and 0.93% of the *C. rhodanensis* J52 genome comprised interspersed repeat sequences and tandem repeat sequences, respectively (Table 2). The circular genome map of *C. rhodanensis* J52 is presented in Figure 1.

### 3.2. C. rhodanensis J52 Genome’s Functional Annotation

Of the 4563 predicted CDSs, 3909 (85.67%) genes, 3486 (76.40%) genes, 3390 (74.29%) genes, 1620 35.50%) genes, 3168 (69.43%) genes and 3056 (66.97%) genes were annotated by the NR, Swiss-Prot, COG, GO and KEGG databases, respectively (Table 3).

**Figure 2:**
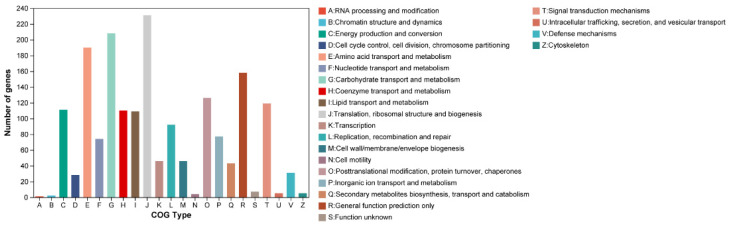
COG classification of protein functions in *C. rhodanensis* J52.

**Figure 3:**
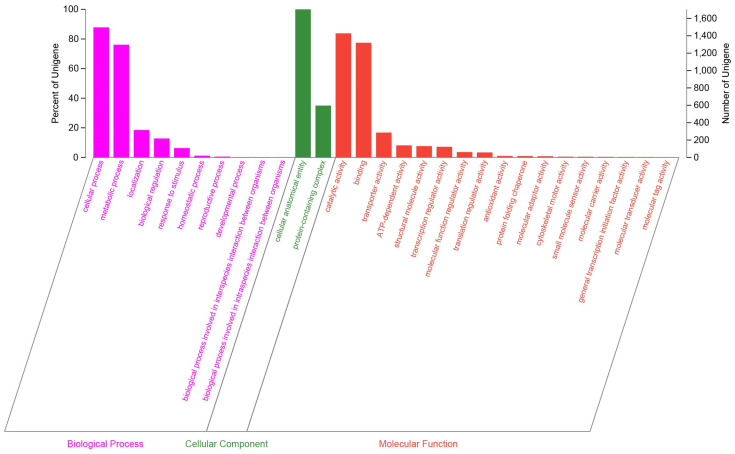
GO classification of gene function annotation in *C. rhodanensis* J52.

**Figure 4:**
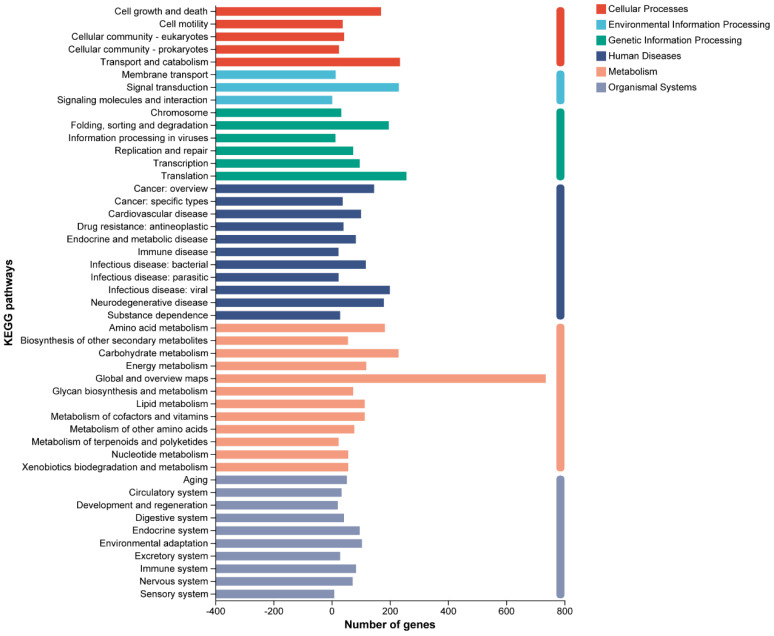
KEGG classification of *C. rhodanensis* J52.

Among the 3909 genes annotated by NR, 97 were classified as putative genes, 597 were hypothetical proteins, and the remaining 3215 were canonical protein-encoding genes. A total of 1620 CDSs were assigned to COGs categories. The functional classes by COGs revealed that *C. rhodanensis* J52 harbors a high proportion of proteins involved in translation, ribosomal structure and biogenesis (COG J) as well as carbohydrate transport and metabolism (COG G), followed by amino acid transport and metabolism (COG E) (Figure 2). In all, 3168 genes were annotated into the GO database. Based on secondary classifications, the largest number of GO-annotated genes was associated with cellular anatomical entities (1703 genes), followed by cellular processes (1496 genes) and catalytic activity (1428 genes) (Figure 3). The majority of the KEGG annotations for *C. rhodanensis* J52 were related to metabolic pathways (Figure 4).

**Figure 5:**
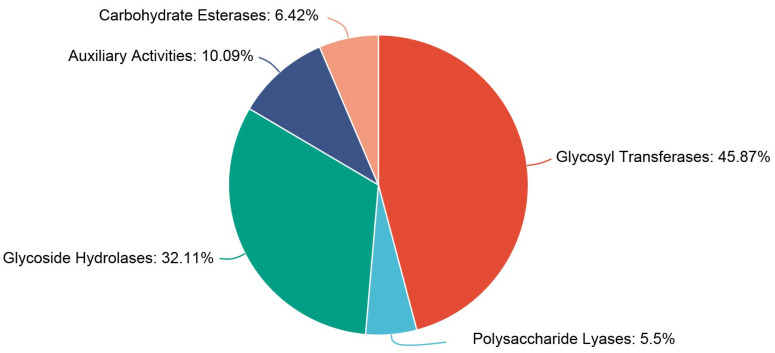
CAZyme classification of protein function in *C. rhodanensis* J52.

Analysis of the *C. rhodanensis* J52 genome identified 109 genes encoding CAZymes, which were classified into five categories: 6 polysaccharide lyases, 7 carbohydrate esterases, 11 auxiliary activities, 35 glycoside hydrolases and 50 glycosyl transferases (Figure 5). Functional annotation of these enzymes indicated that there are four β-glucosidases, three of which are secreted proteins which act outside the cell (Table S3).

### 3.3. Secondary Metabolite Biosynthesis Gene Clusters

**Figure 6:**
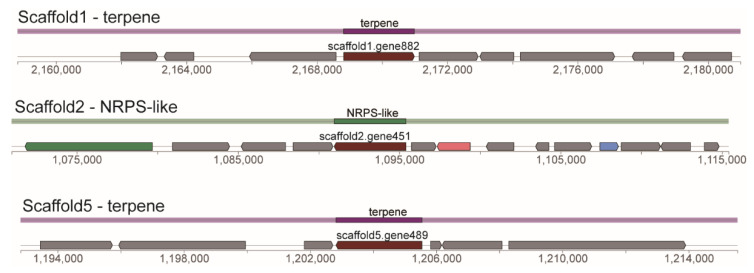
Predicted results of secondary metabolite biosynthesis gene clusters in the *C. rhodanensis* J52.

The genome of *C. rhodanensis* J52 contains three gene clusters: two involved in terpene biosynthesis and one encoding an NPRS-like protein (Figure 6). According to the MIBiG database annotation, these gene clusters are associated with the synthesis of clavaric acid, livipeptin and squalestatin S1 (Table 4).

### 3.4. Genes and Pathways Involved in Flavor Production

#### 3.4.1. Genes Involved in the Production of Flavor

**Figure 7:**
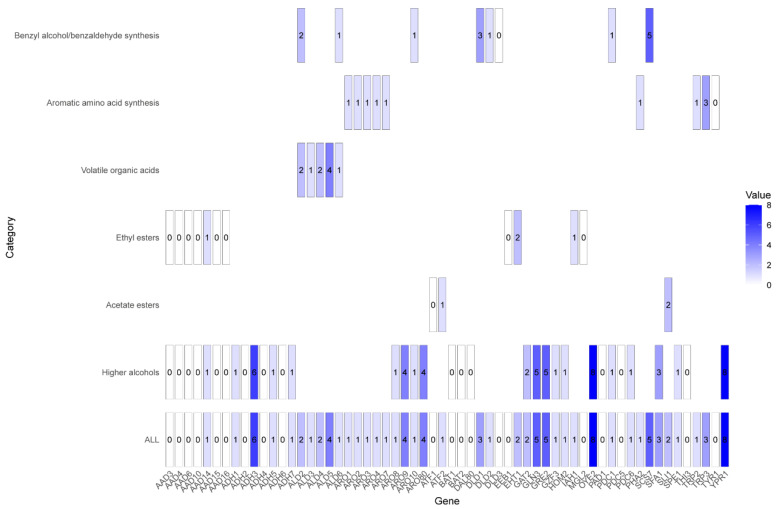
Genes involved in biosynthesis for key flavor compounds in *C. rhodanensis* J52 (refer to Table S1 for detailed descriptions of genes).

The genes involved in flavor biosynthesis in *C. rhodanensis* J52 were identified using BLAST analysis and genome functional annotation. The results indicated that 40 genes with varying copy numbers were implicated in the biosynthesis of six types of flavor substances: higher alcohols, acetate esters, ethyl esters, volatile organic acids, aromatic amino acid synthesis and benzyl alcohol and benzaldehyde (Figure 7). The aryl alcohol dehydrogenase encoded by the *AAD* gene is involved in the formation of higher alcohols and ethyl esters, but this yeast contains only one copy of the *AAD*3 gene. Additionally, the alcohol dehydrogenase encoded by the *ADH* gene is also involved in the formation of higher alcohols.

#### 3.4.2. Predicted Production Pathway of Esters

**Figure 8:**
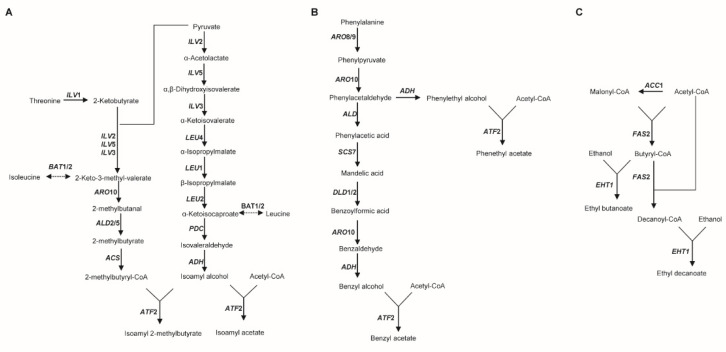
The predicted synthetic pathways for isoamyl acetate, isoamyl 2-methylbutyrate (**A**), benzyl acetate, phenethyl acetate (**B**), ethyl butanoate and ethyl decanoate (**C**) are presented (refer to Table S4 for detailed descriptions of genes).

A previous study demonstrated that fermented peppers prepared with *C. rhodanensis* J52 exhibited significant increases in various flavor compounds compared with the control group without strain inoculation, particularly esters such as isoamyl acetate, isoamyl 2-methylbutyrate, benzyl acetate, phenylethyl acetate, ethyl butyrate and ethyl decanoate [8]. Combining the annotation of CDSs in the *C. rhodanensis* J52 genome and KEGG pathway analysis allowed for prediction of the synthesis pathway for these significantly increased ester compounds. As shown in Figure 8, *C. rhodanensis* J52 mainly produces isoamyl acetate and isoamyl 2-methylbutyrate through the pyruvate metabolic pathway. Due to the absence of *BAT1* and *BAT2*, isoamyl acetate and isoamyl 2-methylbutyrate cannot be synthesized via the Ehrlich pathway from leucine and isoleucine. Additionally, threonine can be metabolized to produce 2-methylbutyrate, which ultimately yields isoamyl 2-methylbutyrate. The yeast may also generate benzyl acetate and phenylethyl acetate through the phenylalanine metabolic pathway. Ethyl butanoate and ethyl decanoate may be formed from the intermediate metabolites butyryl-CoA and decanoyl-CoA in the fatty acid metabolism pathway, catalyzed by alcohol acetyltransferase (encoded by *ATF2*), with ethanol.

### 3.5. Prediction of Bio-Active Potential: Selenoproteins

**Figure 9:**
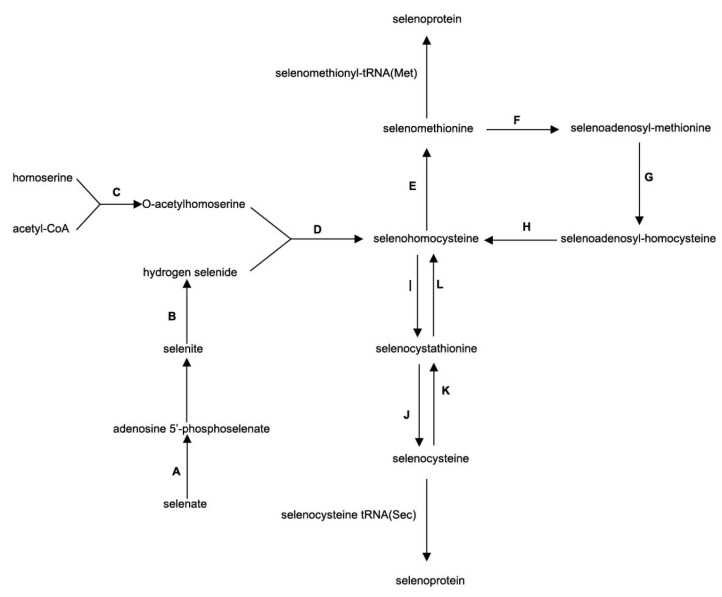
Predicted biosynthesis pathway of selenoproteins in *C. rhodanensis* J52. (A) sulfate adenylyltransferase (gene0882); (B) thioredoxin reductase (gene2327); (C) homoserine O-acetyltransferase (gene1172); (D) homocysteine/cysteine synthase (gene3423); (E) 5-methyltetrahydropteroyltriglutamate--homocysteine methyltransferase (gene3741); (F) S-adenosylmethionine synthase (gene2078); (G) sphingolipid C9-methyltransferase (gene4377); (H) adenosylhomocysteinase (gene1699); (I) cystathionine β-synthase (gene4292); (J) cystathionine γ-lyase (gene3553, gene4075); (K) cystathionine γ-synthase (gene1473); (L) cystathionine β-lyase (gene4075).

Analysis of the non-coding RNAs within the genome of *C. rhodanensis* J52 revealed that in addition to the 20 standard amino acid tRNAs, the yeast also possesses an additional tRNA for selenocysteine (Table S2). This suggests that the yeast has the potential to biosynthesize selenoproteins. Furthermore, based on the annotation analysis of CDSs, it was found that the yeast genome harbors genes homologous to a series of genes necessary for the biosynthesis of selenoproteins, as shown in Figure 9.

### 3.6. Adaptation to the Fermentation Environment

During industrial fermentation, yeast is subjected to various stresses, including osmotic pressures, ethanol stress, oxidative stress, low pH levels and heat stress. The strain possesses genes which enable it to cope with these stresses, which is fundamental to its survival and vitality in such an environment. Functional annotations from the NR and Swiss-Prot databases indicate that the genome of *C. rhodanensis* J52 contains genes involved in adaptation to the fermentation environment: 19 genes related to osmotic pressures, 5 genes associated with ethanol stress, 53 genes responsive to oxidative stress, 13 genes related to low pH levels, 1 gene responsive to SO_2_, and 11 genes encoding heat shock proteins (Table S5).

## 4. Discussion

Non-*Saccharomyces* yeasts have attracted attention due to their high production of esters, which can impart distinctive flavors to fermented foods. *Cyberlindnera* species have been shown to produce elevated levels of acetate, isopentyl, ethyl, and 2-phenylethyl esters, positioning them as promising candidates for industrial applications [18]. In this study, we sequenced and analyzed the genome of *C. rhodanensis* J52 to enhance the utilization of this strain. The genome assembly revealed a length of 13,736,238 bp with a GC content of 51.49%, encoding 4563 proteins. This is comparable to the genome size and GC content of *C. rhodanensis* NRRL Y-7854 (13,910,146 bp; GC content: 51.5%; accession: GCA_030569395.1). Notably, a comprehensive search of the NCBI and SGD databases revealed these as the sole genomic data available for *C. rhodanensis*. In light of these findings, sequencing additional *C. rhodanensis* strains is imperative to obtain comprehensive genomic information, which is essential for a thorough understanding of this species and its optimal utilization.

Our previous studies demonstrated that the involvement of *C. rhodanensis* J52 in fermentation significantly correlates with the content of flavor compounds, particularly esters such as isoamyl acetate [8]. BLAST analysis revealed that the CDSs of *C. rhodanensis* J52 exhibit homology with genes associated with synthetic flavors in *S. cerevisiae* (Figure 7). Genes encoding enzymes involved in the metabolic pathways for synthesizing these flavor compounds—such as ethyl acetate, isopentyl acetate, benzyl acetate, phenylethyl acetate and ethyl decanoate—were identified in the genome of this yeast. This finding further confirms the genetic capacity of yeast to enhance flavor in fermented foods. Higher alcohols are also primary flavor compounds in fermented foods and serve as a substrate for ester synthesis. The formation of higher alcohols can occur via either the amino acid metabolic pathway or the pyruvate metabolic pathway [13,19,20]. As illustrated in Figure 8A, isoamyl alcohol is formed through the transamination, decarboxylation and reduction reactions of leucine, but no transaminase (encoded by *BAT1/2*) was identified in the yeast genome, indicating that isoamyl alcohol is synthesized through the pyruvate metabolic pathway, whereas yeasts containing the *BAT1/2* gene can utilize leucine and valine as an alternative substrate for isoamyl alcohol production, known as the Ehrlich pathway [20]. Subsequently, isoamyl alcohol reacts with acetyl CoA under the action of AATases encoded by *ATF* genes to form isoamyl acetate [19]. Furthermore, studies indicate that this yeast possesses the *ATF2* gene but lacks the *BAT2* gene, which may enhance the production of acetate esters, including isoamyl acetate [21]. The yeast also synthesizes isoamyl 2-methylbutyrate, with 2-methylbutyryl-CoA being metabolized via pyruvate or threonine in the presence of various enzymes [20]. However, this compound cannot be synthesized from isoleucine metabolism due to the absence of *BAT1/2*. The synthetic pathways for benzyl acetate and phenethyl acetate are complete in *C. rhodanensis* J52, as indicated by the phenylalanine metabolic pathway (ko00360) in the yeast and research conducted by Martin et al. [22]. These pathways are illustrated in Figure 8B. Additionally, Figure 7 shows that the yeast contains the genes necessary for synthesizing benzyl acetate and phenethyl acetate. The formation of acetate esters, which impart fruity and floral aromas, is largely dependent on the AATases encoded by the *ATF* gene [13]. Furthermore, AATases exhibit broad specificity and can recognize various acyl-CoA molecules and other substrate alcohols [23]. It is precisely due to the presence of AATases that the yeast possesses the ability to synthesize a diverse range of esters. Figure 8C also depicts the synthetic pathways for ethyl butanoate and ethyl decanoate, which have significantly increased. Both butyryl-CoA and capryl-CoA may be derived from the fatty acid metabolic pathway (ko00061). The combination of butyryl-CoA and capryl-CoA with ethanol to biosynthesize the corresponding esters may be catalyzed by acyl-coenzyme A: ethanol O-acyltransferase (encoded by *EEB1* or *EHT1*) [13,24]. In terms of flavor formation, the yeast also contains β-glucosidase, which catalyzes the hydrolysis of glucosidase-bound aromatic precursors, thereby releasing aromatic compounds [25,26]. The non-*Saccharomyces* yeast *C. rhodanensis* DK was also demonstrated to exhibit extracellular β-glucosidase production [5]. Notably, the yeast has the capability to synthesize selenoproteins (Figure 9). The ability of yeast to synthesise selenoproteins has also been demonstrated in yeasts such as *Kazachstania unispora* and *Pichia kudriavzevii* [27,28]. Selenoproteins have been demonstrated to possess anticancer properties, which include the mitigation of colorectal cancer and esophageal cancer [29,30,31]. Therefore, this yeast can not only enhance the flavor of fermented foods but also provide selenoproteins with anti-cancer properties.

During fermentation, yeast must overcome various stresses, including osmotic stress, ethanol stress, oxidative stress, low pH levels, sulfur dioxide, and ionic and heavy metal stress. In the early stages of fermentation, the fermentation medium contains a high concentration of nutrients, including sugar, which leads to elevated osmotic pressure. To maintain vitality and fermentation, yeast must respond to an environment with high osmotic pressure which may induce internal osmotic imbalance. *Sln1* and *Sho1* are two independent upstream osmosensing mechanisms which specifically activate MAP3K under high osmotic pressure, thereby initiating a series of adaptive responses [32]. Functional annotation of genes indicates that these two genes are present in the *C. rhodanensis* J52 genome (Table S4). Additionally, the yeast contains genes for the synthesis and metabolism of glycerol as well as genes encoding proteins responsible for uptake and excretion, along with genes for trehalose synthesis. The synthesis and metabolism of glycerol and trehalose are primary strategies employed by yeast to adapt to changes in osmotic pressure [32,33]. Therefore, *C. rhodanensis* J52 demonstrated the ability to adapt to high osmotic pressure environments. High concentrations of ethanol adversely affect cellular components, including the phospholipid bilayer, proteins, and other cellular components. The yeast possesses alcohol dehydrogenase and the zinc-binding alcohol dehydrogenase domain-containing protein cipB, which enhance its tolerance to high concentrations of ethanol (Table S5) [34]. Multiple copies of the genes encoding V-type proton ATPase, Na^+^/H^+^ antiporter and H^+^/Cl^−^ exchange transporter 3 in the yeast facilitate adaptation to acidic environments. Previous studies on the co-fermentation of yeast and lactic acid bacteria have also demonstrated that yeast can adapt to low-acid environments [8]. In addition, the *SSU1* gene identified in the yeast genome aids in sulfur dioxide tolerance, indicating the yeast’s potential for wine fermentation [35]. The metal tolerance protein, manganese resistance protein and divalent cation/proton antiporter associated with ionic and heavy metal stress resistance are present in the *C. rhodanensis* J52 genome (Table S5). Heat stress proteins, which maintain balanced physiological functions under various stress conditions, along with other stress-related proteins, are also present in the yeast (Table S5), with all contributing to its ability to respond to diverse stressors. Almost all compounds which induce one type of stress also elicit other stress responses, indicating that stressors do not occur in isolation. When cells face complex environments, multiple genes or signal pathways collaborate to ensure the stability of cellular physiological functions. In addition, the yeast harbors gene coding for killer toxins, which prevents contamination caused by other yeasts during fermentation (Table S6).

## 5. Conclusions

In this study, the genome of *C. rhodanensis* J52, a type of non-*Saccharomyces* yeast with ester-producing capability isolated from edible roses, was sequenced and bioinformatically analyzed. The results indicate that the yeast has a genome size of 13.7 Mb, a GC content of 51.49%, 21 types of tRNAs and 4563 CDSs. These CDSs were annotated using the NR, Swiss-Prot, COG, GO and KEGG database, respectively. Further analysis based on these annotations revealed that this yeast possesses genes involved in the biosynthesis of flavor compounds such as isoamyl acetate, isoamyl 2-methylbutyrate, benzyl acetate, phenethyl acetate, ethyl butanoate and ethyl decanoate, along with the predicted pathways for their synthesis. Additionally, the yeast genome contains the Sec-tRNAsec and a complete metabolic pathway for selenoprotein biosynthesis. Moreover, it has genes related to adaptation to harsh fermentation environments. These findings provide a theoretical foundation for the development and application of *C. rhodanensis* J52 at the genetic level. Future research efforts could focus on exploiting the beneficial biotechnological properties of this yeast strain. Its potential application in the production of selenoproteins, as well as its ability to enhance the flavor of products when mixed with other strains for fermentation, warrant further exploration.

## Figures and Tables

**Table 1:** The genome characteristics of *C. rhodanensis* J52.

Genome Characteristic	J52
Genome size (bp)	13,736,238
Number of scaffolds	8
GC content (%)	51.49
CDSs	4563
Gene average length	2247.05
Number of tRNA gens	175
Number of rRNA gens	33

**Table 2:** The repeated sequences in *C. rhodanensis* J52.

Type	Elements	Number	Length (bp)	Percentage of Sequence (%)
Interspersed repeats	Retroelements	1216	190,990	1.39
	DNA transposons	425	36,821	0.27
	Unclassified	663	392,997	2.86
Tandem repeats	Satellites	24	1700	0.01
	Simple repeat	2508	112,674	0.82
	Low complexity	286	14,168	0.10

**Table 3:** Summary of the statistics of the functional annotation of the CDSs.

Database	Annotated Number	Percent (%)
NR	3909	85.67
Swiss-Prot	3486	76.40
COG	1620	35.50
GO	3168	69.43
KEGG	3056	66.97

**Table 4:** Annotation results of the *C. rhodanensis* J52 secondary metabolic synthesis gene cluster based on MIBiG.

Query	Reference	Compound	BLAST Score
scaffold1.gene882	BGC0001248	clavaric acid	610.0
Scaffold2.gene451	BGC0001168	livipeptin	344.0
Scaffold5.gene489	BGC0001839	squalestatin S1	387.0

## Data Availability

The original contributions presented in the study are included in the article/Supplementary Materials, further inquiries can be directed to the corresponding author.

## Supplementary Materials

The following supporting information can be downloaded at https://www.mdpi.com/article/10.3390/jof11020135/s1.
