# Supplementary material for: Genome Sequencing, Assembly, and Characterization of Cyberlindnera rhodanensis J52 as a Non-Saccharomyces Yeast with Ester-Enhancing Potential
Source: J Fungi (Basel). 2025 Feb 11;11(2):135. doi: 10.3390/jof11020135 (PMC11856183; doi:10.3390/jof11020135)
Supplement: Supplementary file 1 [file jof-11-00135-s001.zip › Supplementary Table S2.pdf]

Table S2. tRNA specific types and copy number statistics.

tRNAs type	Copy	tRNAs type	Copy
Ala	11	Lys	10
Arg	11	Met	8
Asn	6	Phe	6
Asp	9	Pro	6
Cys	3	SeC	1
Gln	6	Ser	13
Glu	11	Thr	12
Gly	12	Trp	4
His	4	Tyr	4
Ile	9	Val	12
Leu	17		
